# Programmed cell death 1 inhibitor plus chemotherapy vs. chemotherapy in advanced drive-gene-negative non-small-cell lung cancer patients: A real-world study

**DOI:** 10.3389/fsurg.2022.954490

**Published:** 2022-09-01

**Authors:** Ying Li, Peng Yang, Xiao Zhou, Xuefeng Yang, Shijie Wu

**Affiliations:** ^1^ Respiratory Medicine, Daqing Oilfield General Hospital, Daqing, China; ^2^ Department of Cardiothoracic Surgery, Daqing Oilfield General Hospital, Daqing, China; ^3^ Department of Oncology, Daqing Oilfield General Hospital, Daqing, China

**Keywords:** non-small-cell lung cancer, programmed cell death 1 inhibitor, driver-gene-negative, efficacy, safety

## Abstract

**Objective:**

Programmed cell death 1 (PD-1) inhibitor has been in the market in China for several years, which lacks sufficient domestic evidence regarding its application in lung cancer. Thus, this study intended to assess the treatment outcome and tolerance of PD-1 inhibitor plus chemotherapy in advanced, driver-gene-negative, nonsquamous, non-small-cell lung cancer (NSCLC) patients in a real clinical setting.

**Methods:**

This retrospective cohort study analyzed 68 advanced driver-gene-negative nonsquamous NSCLC patients, among which 38 cases received PD-1 inhibitor plus chemotherapy and 30 cases adopted chemotherapy alone. Disease control rate (DCR), objective response rate (ORR), progression-free survival (PFS), overall survival (OS), and adverse events were reviewed.

**Results:**

Generally, PD-1 inhibitor plus chemotherapy achieved a more satisfying ORR compared with chemotherapy alone (52.6% vs. 30.0%, *P *= 0.061), while the DCR did not vary between PD-1 inhibitor plus chemotherapy and chemotherapy (84.2% vs. 73.3%, *P *= 0.271). Patients receiving PD-1 inhibitor plus chemotherapy exhibited favorable PFS (median: 10.1 vs. 7.1 months, *P *= 0.040) and OS (median: 17.4 vs. 13.9 months, *P *= 0.049) than patients adopting chemotherapy alone. Additionally, after adjustment using multivariable Cox's analyses, PD-1 inhibitor plus chemotherapy (vs. chemotherapy) could independently realize prolonged PFS (*P *= 0.020) and OS (*P *= 0.029). Moreover, the majority of adverse events were manageable; meanwhile, grade 3–4 adverse events included leukopenia (13.2%), neutropenia (13.2%), nausea and vomiting (7.9%), anemia (5.3%), elevated transaminase (5.3%), thrombopenia (2.6%), anorexia (2.6%), peripheral neuropathy (2.6%), and rash (2.6%).

**Conclusion:**

PD-1 inhibitor plus chemotherapy exhibits a better efficacy and equal tolerance compared with chemotherapy alone in advanced driver-gene-negative nonsquamous NSCLC patients.

## Introduction

Non-small-cell lung cancer (NSCLC) acts as one of the most prevalent and fatal malignancies worldwide (1). Patients with advanced-stage NSCLC commonly suffer from a 5-year survival of 14%–29.6%; over decades, novel therapies such as immune therapy, targeted therapy, etc., have largely changed the landscape of advanced NSCLC (2–4). Thanks to the advancement in molecular biology and biotechnology, advanced driver-gene-positive NSCLC patients [such as epidermal growth factor receptor (EGFR), ROS proto-oncogene 1 receptor tyrosine kinase (ROS1), and anaplastic lymphoma kinase (ALK), etc.] could benefit from corresponding targeted therapy and their survival has been improved to some extent (5–7). However, in terms of driver-gene-negative patients, available treatment approaches are still limited, which merely include chemotherapy and its combination with angiogenesis inhibitors or immunotherapy (8, 9). However, exploring possible therapeutic options for advanced driver-gene-negative NSCLC has never stopped.

Programmed cell death 1 (PD-1) inhibitor, a recently introduced therapy that breaks the PD-1/PD ligand 1 (PD-L1) linkage, is widely used in numerous cancer treatments (10, 11). Typically, some trials disclose that PD-1 inhibitor has been applied to treat advanced driver-gene-negative nonsquamous NSCLC: one trial discloses that camrelizumab plus carboplatin and pemetrexed prolonged the progression-free survival (PFS) of advanced drive-gene-negative nonsquamous NSCLC patients (12). Similarly, another trial reports that tislelizumab plus carboplatin or cisplatin could realize a relatively favorable response rate, response duration, and PFS in patients with advanced drive-gene-negative nonsquamous NSCLC (13). Beyond that, the combination of pembrolizumab with pemetrexed and platinum-based drugs could contribute to a prolonged PFS and overall survival (OS) for advanced drive-gene-negative nonsquamous NSCLC patients (14).

However, few studies in the real clinical setting have been carried out in China concerning the treatment efficacy of the PD-1 inhibitor in these patients. Hence, the current real-world study aimed to further explore the treatment outcome and tolerance of PD-1 inhibitor plus chemotherapy in patients with advanced drive-gene-negative nonsquamous NSCLC.

## Methods

### Patients

This was a retrospective cohort study, which reviewed 68 advanced driver-gene-negative nonsquamous NSCLC patients who underwent first-line chemotherapy with or without PD-1 inhibitor from April 2019 to June 2021. The screening criteria were set as (1) histopathologically confirmed as nonsquamous NSCLC; (2) within 18–85 years old; (3) negative driver genes (EGFR, ALK, and ROS1); (4) tumor–node–metastasis (TNM) stage IIIB, IIIC, or IV; (5) received first-line PD-1 inhibitor plus chemotherapy or received chemotherapy alone; (6) had at least one measurable lesion for response assessment in line to the response evaluation criteria in solid tumors (RECIST) (15); and (7) had available clinical data, radiological images, and follow-up information. The patients who had a history of other primary malignant diseases before diagnosis of NSCLC were ineligible for screening. This study was permitted by the Ethics Committee. Written informed consents were required from the patients or their families.

### Treatment regimens

The treatment strategy was made by the patients’ status and discussion between the clinician and patients. The patients who received PD-1 inhibitor plus chemotherapy were considered as the PD-1 inhibitor plus chemo group (*N* = 38), and the patients who received chemotherapy alone were considered as the chemo group (*N* = 30). In the PD-1 inhibitor plus chemo group, patients received PD-1 inhibitor plus chemotherapy on day 1 of each 3-week cycle for four cycles. Concretely, a total of 14 patients received sintilimab + pemetrexed + cisplatin, 9 received sintilimab + pemetrexed + carboplatin, 9 received camrelizumab + pemetrexed + cisplatin, and 6 received camrelizumab + pemetrexed + carboplatin. In the chemo group, patients received chemotherapy alone on day 1 of each 3-week cycle for four cycles. In detail, a total of 16 patients received pemetrexed + cisplatin and 14 patients received pemetrexed + carboplatin. The administrations for PD-1 inhibitor were as follows: sintilimab was administrated intravenously at a dose of 200 mg; camrelizumab was administrated intravenously at a dose of 200 mg. After the four cycles of PD-1 inhibitor treatment, the PD-1 inhibitor was administrated to the NSCLC patients until disease progression or the occurrence of uncontrollable toxicity. The administrations for chemotherapy were as follows: pemetrexed was administered intravenously at a dose of 500 mg/m^2^; cisplatin was administered intravenously at a dose of 75 mg/m^2^; carboplatin was administered intravenously with an area under the curve of 5–6 mg/ml every minute. Detailed information of the treatment regimen is shown in (Table 1).

**Table 1:** Treatment information.

Items	PD-1 inhibitor plus chemo (N = 38)	Chemo (N = 30)
PD-1 inhibitor, No. (%)		
None	0 (0.0)	30 (100.0)
Sintilimab	23 (60.5)	0 (0.0)
Camrelizumab	15 (39.5)	0 (0.0)
Chemotherapy regimen, No. (%)		
Pemetrexed + cisplatin	23 (60.5)	16 (53.3)
Pemetrexed + carboplatin	15 (39.5)	14 (46.7)
Treatment regimen, No. (%)		
Pemetrexed + cisplatin	0 (0.0)	16 (53.3)
Pemetrexed + carboplatin	0 (0.0)	14 (46.7)
Sintilimab + pemetrexed + cisplatin	14 (36.8)	0 (0.0)
Sintilimab + pemetrexed + carboplatin	9 (23.7)	0 (0.0)
Camrelizumab + pemetrexed + cisplatin	9 (23.7)	0 (0.0)
Camrelizumab + pemetrexed + carboplatin	6 (15.8)	0 (0.0)

PD-1, programmed cell death 1.

### Assessment

Demographics and disease characteristics were obtained. Efficacy assessment after four cycles of chemotherapy (about 3 months) was fulfilled based on radiological images according to the RECIST criteria (15). PFS and OS were determined based on the collected follow-up information, and the final date of follow-up recording was September 2021. For safety evaluation, adverse events occurred during the treatment were collected and classified in line with the Common Terminology Criteria for Adverse Events (V.4.0).

### Statistics

Data were analyzed using SPSS V.24.0 (IBM, United States), and graphs were made using GraphPad Prism V.8.01 (GraphPad Software Inc., United States). The difference in clinical characteristics, treatment response, and adverse events between the two groups was determined by the Chi-square test, Fisher's exact test, Mann–Whitney *U* test, or Student's *t* test. PFS and OS were illustrated using the Kaplan–Meier curve and determined by the log-rank test. Prognostic factor analysis was completed using Cox's proportional hazards regression analysis, and all potential factors were included in the multivariate Cox's regression analysis. *P* < 0.05 was considered significant.

## Results

### Clinical characteristics

Generally, there were no differences in the baseline parameters between the PD-1 inhibitor plus chemo group and the chemo group. In detail, the mean ages were 59.1 ± 8.1 years and 61.4 ± 9.1 years in the PD-1 inhibitor plus chemo group and the chemo group, separately (*P* = 0.280, Table 2). With regard to gender, there were 10 (26.3%) females and 28 (73.7%) males in the PD-1 inhibitor plus chemo group; meanwhile, there were 9 (30.0%) females and 21 (70.0%) males in the chemo group (*P* = 0.737). In terms of histological type, there were 36 (94.7%) and 2 (5.3%) patients with adenocarcinoma (ADC) and large-cell carcinoma (LCC) subtypes in the PD-1 inhibitor plus chemo group; meanwhile, there were 29 (96.7%) and 1 (3.3%) with ADC and LCC subtypes in the chemo group (*P* = 1.000). More detailed information is listed in Table 2.

**Table 2:** Clinical characteristics.

Items	PD-1 inhibitor plus chemo (*N* = 38)	Chemo (*N* = 30)	*P* value
Age (years), mean ± SD	59.1 ± 8.1	61.4 ± 9.1	0.280
Gender, No. (%)			0.737
Female	10 (26.3)	9 (30.0)	
Male	28 (73.7)	21 (70.0)	
Smoke status, No. (%)			0.634
Never	8 (21.1)	7 (23.3)	
Former	20 (52.6)	18 (60.0)	
Current	10 (26.3)	5 (16.7)	
Histological type, No. (%)			1.000
ADC	36 (94.7)	29 (96.7)	
LCC	2 (5.3)	1 (3.3)	
ECOG PS, No. (%)			0.876
0	15 (39.5)	12 (40.0)	
1	22 (57.9)	16 (53.3)	
2	1 (2.6)	2 (6.7)	
TNM stage, No. (%)			0.851
Stage IIIB/C	7 (18.4)	5 (16.7)	
Stage IV	31 (81.6)	25 (83.3)	
Bone metastasis, No. (%)			0.648
No	30 (78.9)	25 (83.3)	
Yes	8 (21.1)	5 (16.7)	
Brain metastasis, No. (%)			0.648
No	30 (78.9)	25 (83.3)	
Yes	8 (21.1)	5 (16.7)	

PD-1, programmed cell death 1; SD, standard deviation; ADC, adenocarcinoma; LCC, large-cell carcinoma; ECOG PS, Eastern Cooperative Oncology Group performance status; TNM, tumor–node–metastasis.

### Treatment response

In the PD-1 inhibitor plus chemo group, there were 1 (2.6%), 19 (50.0%), 12 (31.6%), and 6 (15.8%) patients reaching complete response (CR), partial response (PR), stable disease (SD), and progressive disease (PD), respectively; in the chemo group, there were 0 (0.0%), 9 (30.0%), 13 (43.3%), and 8 (26.7%) patients reaching CR, PR, SD, and PD, separately (Table 3). Meanwhile, the objective response rate (ORR) was 52.6% and 30.0% in the PD-1 inhibitor plus chemo group and the chemo group, separately. Additionally, the disease control rate (DCR) was 84.2% and 73.3% in the PD-1 inhibitor plus chemo group and the chemo group, respectively. Collectively, there was a trend that the PD-1 inhibitor plus chemo group had a numerically better ORR (*P* = 0.061) compared with the chemo group, while this finding was without statistical significance. However, the DCR was of no difference between the two groups (*P* = 0.271).

**Table 3:** Treatment response.

Items	PD-1 inhibitor plus chemo (*N* = 38)	Chemo (*N* = 30)	*P* value
Overall response, No. (%)			0.059
CR	1 (2.6)	0 (0.0)	
PR	19 (50.0)	9 (30.0)	
SD	12 (31.6)	13 (43.3)	
PD	6 (15.8)	8 (26.7)	
ORR (CR + PR), No. (%)	20 (52.6)	9 (30.0)	0.061
DCR (CR + PR + SD), No. (%)	32 (84.2)	22 (73.3)	0.271

PD-1, programmed cell death 1; CR, complete response; PR, partial response; SD, stable disease; PD, progressive disease; ORR, objective response rate; DCR, disease control rate.

### Survival

**Figure 1:**
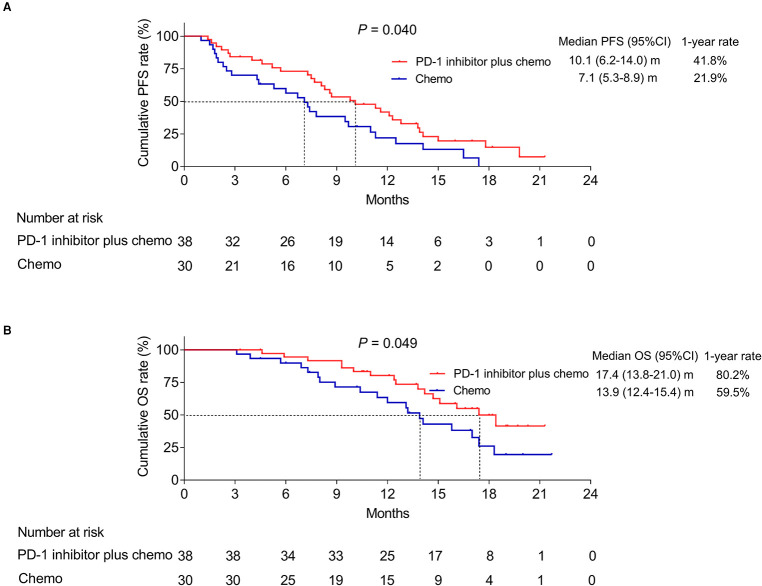
Survival profile. PFS (**A**) and OS (**B**) of advanced driver-gene-negative nonsquamous NSCLC patients adopting PD-1 inhibitor plus chemotherapy and chemotherapy alone.

The mean and median value of the follow-up was 12.9 and 13.5 months with the range of 3.1–21.7 months. Until the last follow-up date by September 2021, in the PD-1 inhibitor plus chemo group, 30 patients got progressive disease and 16 patients died; meanwhile, in the chemo group, 26 patients got progressive disease and 19 patients died. Interestingly, it was disclosed that the PD-1 inhibitor plus chemo group had a longer PFS compared to the chemo group (*P* = 0.040, Figure 1A). In detail, the median PFS was 10.1 [95% confidence interval (CI): 6.2–14.0] months with a 1-year PFS rate of 41.8% in the PD-1 inhibitor plus chemo group and 7.1 (95%CI: 5.3–8.9) months with a 1-year PFS rate of 21.9% in the chemo group.

In addition, the PD-1 inhibitor plus chemo group had a longer OS compared to the chemo group (*P* = 0.049, Figure 1B). In detail, the median OS was 17.4 (95%CI: 13.8–21.0) months with a 1-year OS rate of 80.2% in the PD-1 inhibitor plus chemo group and 13.9 (95%CI: 12.4–15.4) months with a 1-year OS rate of 59.5% in the chemo group, respectively.

**Figure 2:**
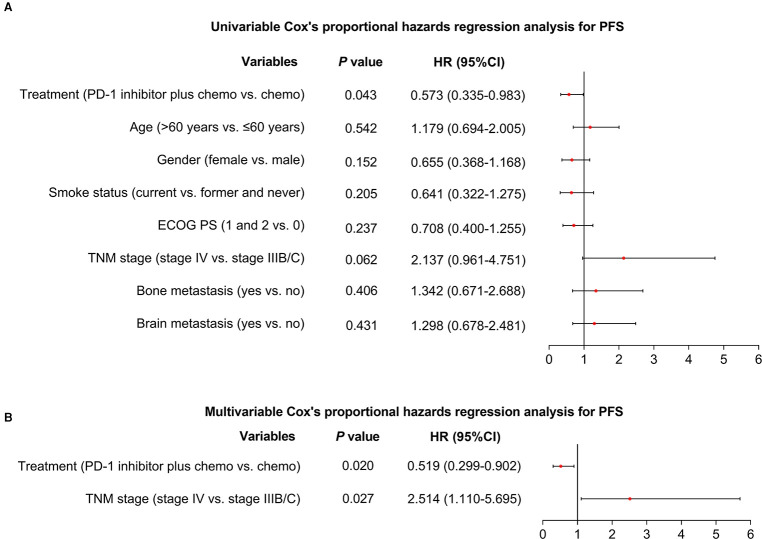
Factors affecting PFS. Univariable (**A**) and multivariable (**B**) Cox's proportional hazards regression analysis for PFS in advanced driver-gene-negative nonsquamous NSCLC patients.

**Figure 3:**
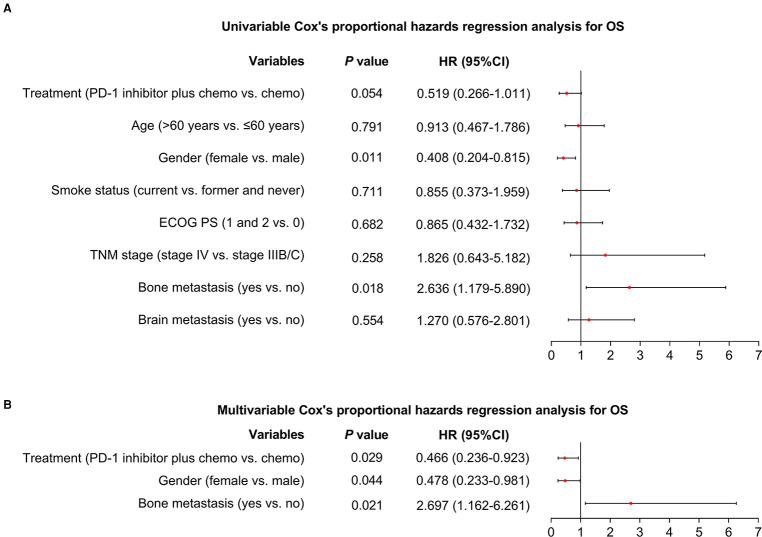
Factors affecting OS. Univariable (**A**) and multivariable (**B**) Cox's proportional hazards regression analysis for OS in advanced driver-gene-negative nonsquamous NSCLC patients.

Additionally, after the adjustment by the multivariate Cox's proportional hazard regression analyses, PD-1 inhibitor plus chemotherapy (vs. chemotherapy alone) could independently realize a longer PFS [hazard ratio (HR) = 0.519 (95%CI: 0.299–0.902), *P* = 0.020, Figures 2A,B] and OS [HR = 0.466 (95%CI: 0.236–0.923), *P* = 0.029, Figures 3A,B].

Apart from that, the subgroup analysis in PD-1 inhibitor plus chemo group was conducted, which showed that the PFS (*P *= 0.084, Supplementary Figure S1A) and OS (*P *= 0.289, Supplementary Figure S1B) were of no difference among the sintilimab + pemetrexed + cisplatin, sintilimab + pemetrexed + carboplatin, camrelizumab + pemetrexed + cisplatin, and camrelizumab + pemetrexed + carboplatin subgroups. In addition, the PFS (*P *= 0.899, Supplementary Figure S2A) and OS (*P *= 0.791, Supplementary Figure S2B) were also not different between sintilimab plus chemotherapy and camrelizumab plus chemotherapy subgroups.

### Adverse events

Generally, the adverse events were manageable; meanwhile, these adverse events were not varied between the two groups (all *P *>* *0.05, Table 4). In the PD-1 inhibitor plus chemo group, most adverse events were at grade 1–2; meanwhile, the main grade 3–4 hematological adverse events included leukopenia (13.2%), neutropenia (13.2%), anemia (5.3%), and thrombopenia (2.6%). Additionally, grade 3–4 nonhematological adverse events included nausea and vomiting (7.9%), elevated transaminase (5.3%), anorexia (2.6%), rash (2.6%), and peripheral neuropathy (2.6%). More detailed information is listed in Table 4.

**Table 4:** Adverse events.

Adverse events	PD-1 inhibitor plus chemo (*N* = 38)			Chemo (*N* = 30)			*P* value*
	Total	Grade 1–2	Grade 3–4	Total	Grade 1–2	Grade 3–4	
Hematological adverse events							
Leukopenia, No. (%)	14 (36.8)	9 (23.7)	5 (13.2)	9 (30.0)	5 (16.7)	4 (13.3)	0.554
Neutropenia, No. (%)	13 (34.2)	8 (21.1)	5 (13.2)	9 (30.0)	8 (26.7)	1 (3.3)	0.712
Anemia, No. (%)	11 (28.9)	9 (23.7)	2 (5.3)	8 (26.7)	7 (23.3)	1 (3.3)	0.835
Thrombopenia, No. (%)	8 (21.1)	7 (18.4)	1 (2.6)	6 (20.0)	6 (20.0)	0 (0.0)	0.915
Nonhematological adverse events							
Fatigue, No. (%)	17 (44.7)	17 (44.7)	0 (0.0)	11 (36.7)	11 (36.7)	0 (0.0)	0.502
Peripheral neuropathy, No. (%)	14 (36.8)	13 (34.2)	1 (2.6)	12 (40.0)	12 (40.0)	0 (0.0)	0.790
Elevated transaminase, No. (%)	12 (31.6)	10 (26.3)	2 (5.3)	9 (30.0)	8 (26.7)	1 (3.3)	0.889
Nausea and vomiting, No. (%)	12 (31.6)	9 (23.7)	3 (7.9)	8 (26.7)	7 (23.3)	1 (3.3)	0.659
Alopecia, No. (%)	11 (28.9)	11 (28.9)	0 (0.0)	8 (26.7)	8 (26.7)	0 (0.0)	0.835
Diarrhea, No. (%)	8 (21.1)	8 (21.1)	0 (0.0)	7 (23.3)	7 (23.3)	0 (0.0)	0.822
Anorexia, No. (%)	8 (21.1)	7 (18.4)	1 (2.6)	6 (20.0)	5 (16.7)	1 (3.3)	0.915
Increased blood pressure, No. (%)	8 (21.1)	8 (21.1)	0 (0.0)	5 (16.7)	5 (16.7)	0 (0.0)	0.648
Elevated bilirubin, No. (%)	8 (21.1)	8 (21.1)	0 (0.0)	4 (13.3)	4 (13.3)	0 (0.0)	0.407
Rash, No. (%)	6 (15.8)	5 (13.2)	1 (2.6)	5 (16.7)	5 (16.7)	0 (0.0)	1.000
RCCEP, No. (%)	6 (15.8)	6 (15.8)	0 (0.0)	0 (0.0)	0 (0.0)	0 (0.0)	—
Constipation, No. (%)	4 (10.5)	4 (10.5)	0 (0.0)	4 (13.3)	4 (13.3)	0 (0.0)	0.724

PD-1, programmed cell death 1; RCCEP, reactive cutaneous capillary endothelial proliferation.

*Comparison of total adverse events between two groups.

## Discussion

So far, several studies evaluate the treatment response of PD-1 inhibitor in advanced, driver-gene-negative NSCLC patients. One study reveals that patients receiving tislelizumab plus chemotherapy could realize an increased ORR compared with chemotherapy alone (57.4% vs. 36.9%) in advanced driver-gene-negative nonsquamous NSCLC patients (13). Another study illustrates that sintilimab plus chemotherapy could also elevate the ORR compared with chemotherapy alone (51.9% vs. 29.8%) in these patients (16). In the current study, the PD-1 inhibitor plus chemotherapy could realize a numerically increased ORR compared with placebo chemotherapy (52.6% vs. 30.0%) in these patients. Possible explanations would be that (1) PD-1 inhibitor enhances the activity of the tumor-infiltrating lymphocytes and promotes cytotoxic secretions for antitumor immune responses; thus, PD-1 inhibitor could exhibit a satisfying treatment efficacy (17). (2) PD-1 inhibitor and chemotherapy have a synergistic efficacy, thus the PD-1 inhibitor plus chemotherapy would realize better treatment outcomes compared to the chemotherapy (18).

It has been also illustrated that PD-1 inhibitor plus chemotherapy could prolong the survival for advanced drive-gene-negative nonsquamous NSCLC patients: a previous study discloses that sintilimab plus chemotherapy could realize a median PFS of 8.9 months in advanced drive-gene-negative nonsquamous NSCLC patients (16). In addition, another study illuminates that tislelizumab plus chemotherapy could realize a longer PFS (median: 9.7 vs. 7.6 months) compared with chemotherapy alone in these patients (13). Herein, our study also exhibited a similar result: the PD-1 inhibitor plus chemotherapy exhibited a longer PFS and OS compared with the chemotherapy alone in these patients. In detail, PD-1 inhibitor plus chemotherapy realized median PFS and OS of 10.1 and 17.4 months, respectively in advanced driver-gene-negative nonsquamous NSCLC patients. These results above disclose that PD-1 inhibitor plus chemotherapy exhibits an acceptable treatment efficacy in advanced nonsquamous NSCLC patients. Possible explanations could be that (1) PD-1 inhibitor plus chemotherapy leads to a numerically better ORR; therefore, this difference could result in a prolonged survival profile. (2) PD-1 inhibitor synergizes with chemotherapy and therefore improves the survival profile for advanced NSCLC patients (18). In addition, this study also observed that the female patients might achieve a prolonged OS, while those patients with bone metastasis could reach a shorter OS. These findings suggested that the clinicians should make more effort and give more attention in treating male or bone metastasis NSCLC patients because their survival was inherently unsatisfying.

Adverse events of PD-1 inhibitor plus chemotherapy in advanced NSCLC patients are also critical concerns to its application. In addition, since the PD-1 inhibitor acts with an antitumor role through inhibiting the PD-1 expression in T cells, thus its hematological toxicity is non-neglectable: a previous study reveals that the occurrence of grade 3–4 adverse events is low, which mainly includes leukopenia, neutropenia, pneumonitis, cholangitis, neuropathy, and hypokalemia (13). The present study also showed a similar result: the adverse events were manageable; meanwhile, these adverse events were not varied between the two groups. The adverse events were relatively manageable and most adverse events were grade 1–2. In addition, grade 3–4 adverse events included leukopenia (13.2%), neutropenia (13.2%), nausea and vomiting (7.9%), anemia (5.3%), elevated transaminase (5.3%), thrombopenia (2.6%), anorexia (2.6%), peripheral neuropathy (2.6%), and rash (2.6%). These findings disclose that PD-1 inhibitor plus chemotherapy exhibits an acceptable tolerance in advanced driver-gene-negative NSCLC patients. Reactive cutaneous capillary endothelial proliferation (RCCEP) is a common adverse event that is reported during camrelizumab treatment. In our study, we found that the incidence of RCCEP in PD-1 inhibitor plus chemo was 15.8%. Therefore, this finding indicated that clinicians should notice the RCCEP occurrence and deal with this adverse event suitably.

Despite the innovation of the current study, several limitations still existed: (1) The present study was a single-center study; thus, patients’ selection bias might exist here due to the regional restriction. (2) The present study merely enrolled 68 advanced nonsquamous NSCLC patients; therefore, the statistical power might be limited. (3) This study enrolled nonsquamous NSCLC patients, while the efficacy of PD-1 inhibitor plus chemotherapy in squamous NSCLC patients was not evaluated in the present study. (4) The PD-L1 expression was not determined in this study; further study could determine the impact of PD-L1 expression on the survival of NSCLC patients.

Conclusively, PD-1 inhibitor plus chemotherapy exhibits a better efficacy and equal tolerance compared with chemotherapy alone in advanced driver-gene-negative nonsquamous NSCLC patients.

## Data Availability

The original contributions presented in the study are included in the article/Supplementary Material, further inquiries can be directed to the corresponding author.
