# Supplementary figures and images for: Programmed cell death 1 inhibitor plus chemotherapy vs. chemotherapy in advanced drive-gene-negative non-small-cell lung cancer patients: A real-world study
Source: Front Surg. 2022 Sep 1;9:954490. doi: 10.3389/fsurg.2022.954490 (PMC9475215; doi:10.3389/fsurg.2022.954490)

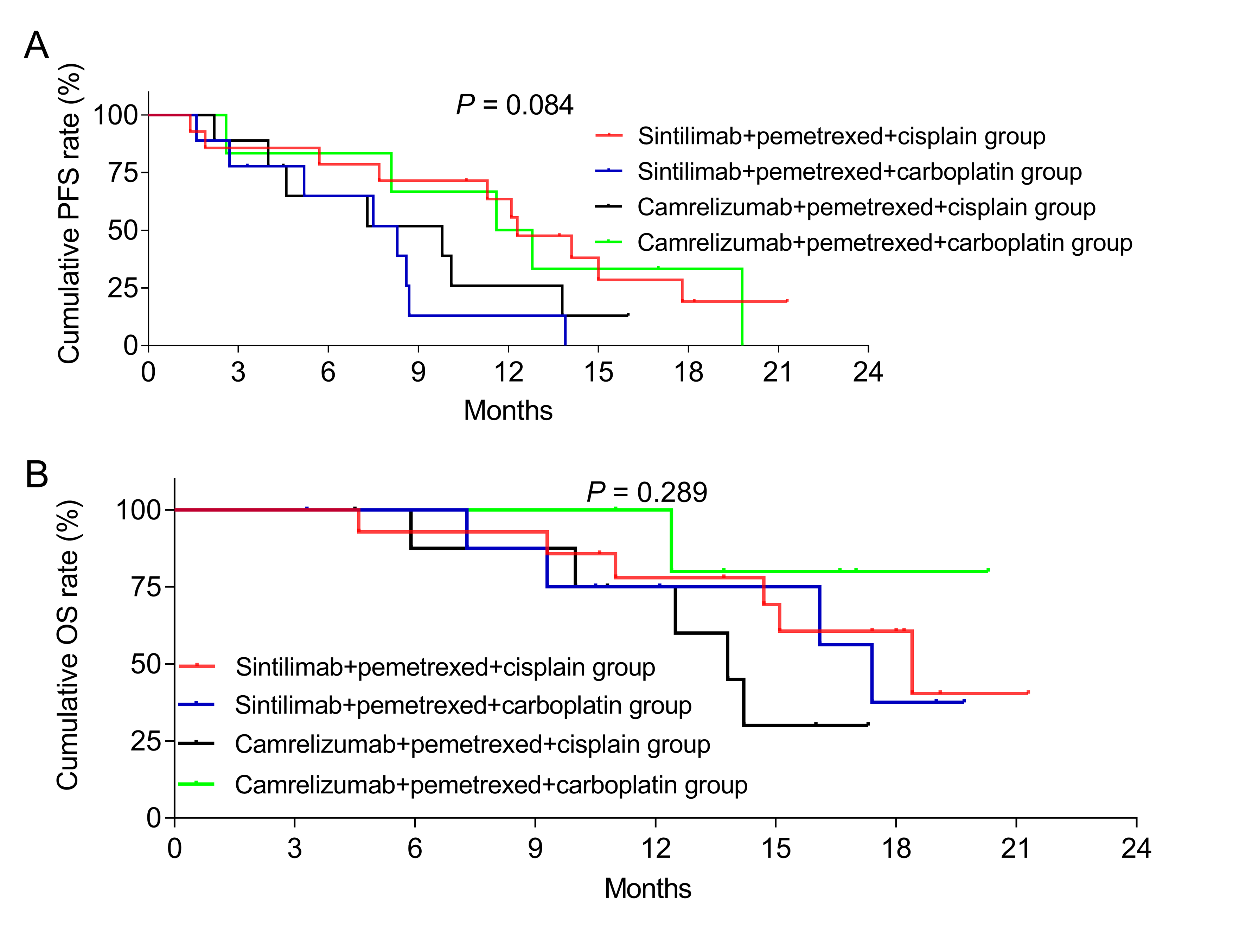

Supplement: Supplementary file 1 [file Image_1_v1.tif]

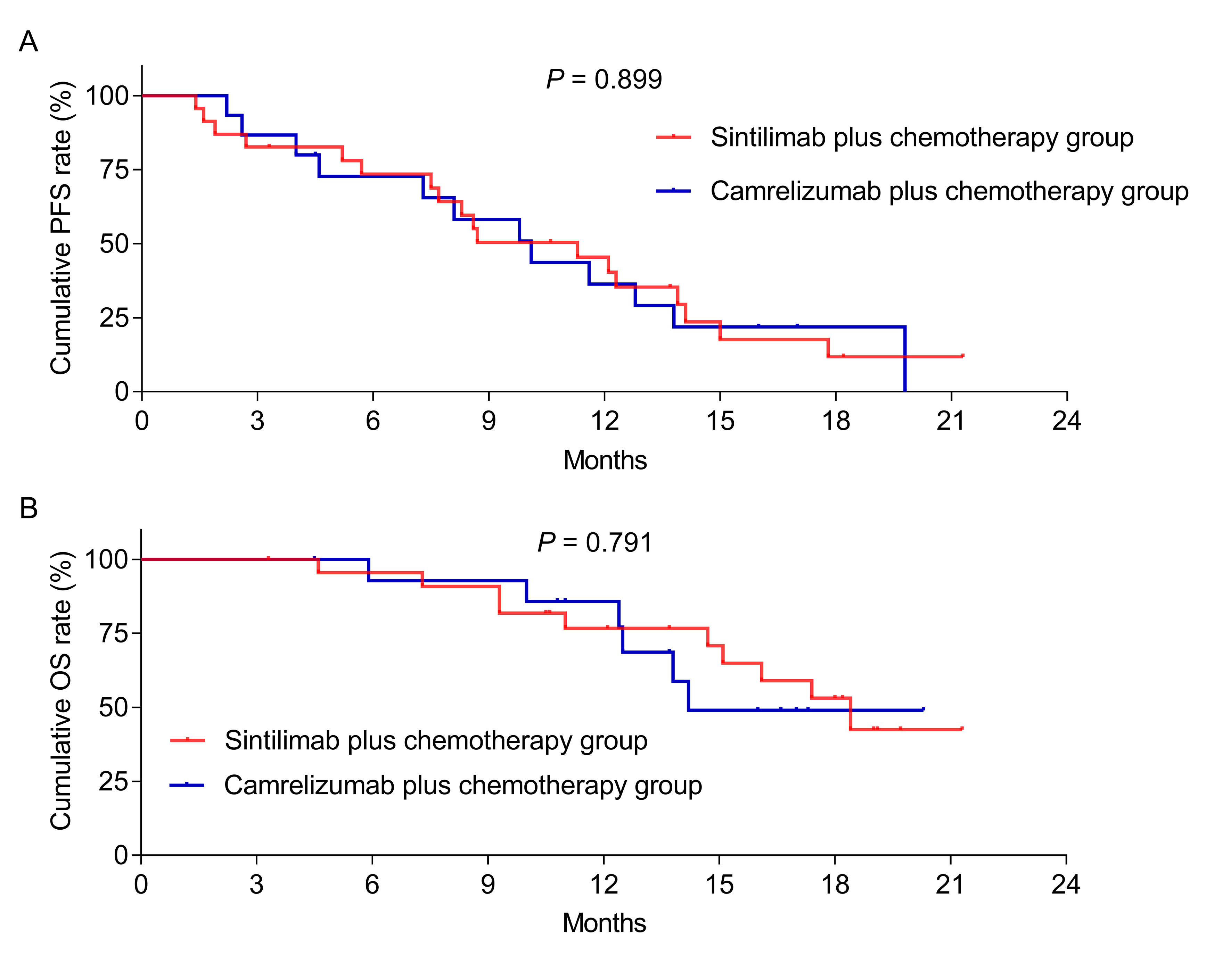

Supplement: Supplementary file 2 [file Image_2_v1.tif]
